# The relationship between COVID-19-induced death thoughts and depression during a national lockdown

**DOI:** 10.1177/13591053211067102

**Published:** 2021-12-29

**Authors:** Samuel Fairlamb

**Affiliations:** Royal Holloway, University of London, UK

**Keywords:** COVID-19, depression, self-esteem, terror management, well-being

## Abstract

Evidence suggests that the COVID-19 pandemic has increased rates of depression worldwide. Many factors have been identified to relate to this increase depression, but according to Terror Management Theory, the heightened awareness of death during the pandemic has the potential to increase depression for those with low self-esteem. This hypothesis was examined in a U.K. sample during the first national lockdown where depression, self-esteem and death-thought accessibility (DTA) were measured, and a COVID Index (COVID-19 cases and deaths on date of participation) was produced. The COVID Index was positively related to DTA. Additionally, DTA mediated the relationship between the COVID Index and depression scores when participants had low levels of self-esteem. These findings suggest that heightened existential concerns may be a neglected factor increasing depression during the pandemic.

Increases in mental health difficulty worldwide have been identified since the beginning of the pandemic (Bueno-Notivol et al., 2021; Torales et al., 2020). The limits to people’s freedom by imposed lockdowns, quarantines and restrictions on travel; increasing social isolation and loneliness; and increasing career and financial insecurity have been identified as predictors of depression (e.g. Rudenstine et al., 2021; Ustun, 2021).

While all of these issues have likely contributed towards an increase in depression, the pandemic as a timely and potent reminder of our transient nature (Courtney et al., 2020), has been a neglected factor in this increase in depression. The present research provides novel evidence that heightened existential concerns throughout lockdown are related to increases in depressive symptomology.

According to Terror Management Theory (TMT; Greenberg et al., 1986), humans are motivated to stay alive, yet are acutely aware of the fact they will die. This juxtaposition poses a powerful psychological threat that can be inherently demotivating (Hayes et al., 2016), and might be a breeding ground for feelings of hopelessness and depression (Becker, 1973; Yalom, 2008). However, according to TMT, such feelings are kept at bay by a sense that one possesses a sense of self-esteem, which is achieved by feeling like one is living up to consensually validated cultural standards of conduct. Therefore, TMT stipulates the necessary conditions for sound mental health and why those conditions are necessary (Juhl, 2019).

Support for the theory stems from the *mortality salience* hypothesis that proposes if a psychological structure (i.e. self-esteem) helps to manage death concerns, then heightening the awareness of death should increase the need to rely on these psychological structures (Pyszczynski et al., 2015). Studies have generally supported this assertion across various national contexts and cultural settings (Burke et al., 2010).

Another line of evidence of the theory is the *death thought accessibility* (DTA) hypothesis (Pyszczynski et al., 2015). This hypothesis states that if certain structures (i.e. self-esteem) manage thoughts of death then threatening these structures should increase the accessibility of death-related thoughts. Typically, DTA is measured using a word-stem completion task whereby some words can be completed in a death manner (e.g. KI _ _ E D can be *killed* or *kissed*). Supporting this hypothesis, Hayes et al. (2008) found that threatening one’s self-esteem increased DTA but did not increase negative thoughts.

Although TMT has primarily been applied to understand a range of social phenomena (Pyszczynski et al., 2015), the theory also provides an account regarding psychological well-being (Juhl, 2019). It argues that self-worth is critical in preventing the awareness of death from undermining one’s well-being. For example, research has identified that mortality salience can reduce well-being (Routledge et al., 2010), and increase depressive mood after reflecting on failure (Hayes et al., 2016) for those with low self-esteem. In addition, DTA predicts depressive symptoms for those who lack self-esteem (Fairlamb and Juhl, 2020).

If the theory’s assertions about the role of death awareness in psychological well-being are correct, then it would imply that situations that can increase the accessibility of death-related thoughts would have the potential to increase depression for those who lack self-esteem. A global pandemic might be one such scenario where death thoughts are generally more accessible for a myriad of reasons. Most directly, thoughts of death might be more salient because the pandemic has increased people’s anxiety and fear regarding the threat to their physical health (Jungmann and Witthöft, 2020), as well as the threat to loved ones. Health threats such as COVID-19 have the potential to increase DTA (Arndt and Goldenberg, 2017). More generally, increased exposure to death-related themes in the news can increase DTA (e.g. Das et al., 2009), and research suggests that exposure to COVID-19 news is associated with increased mental health difficulty (Gao et al., 2020).

Of course, DTA is also likely to fluctuate temporally in the pandemic. This is because the threat of COVID-19 is likely to be more visceral in the mind of the public when cases and mortality rates are high. For example, people are likely to be reminded more often of the deadly threat of the virus, and the need to engage in virus mitigating behaviour, which should heighten DTA.

## The present study

Shortly before the pandemic began, the author of this research was conducting a study to examine DTA predicting increased depression at low levels of self-esteem (*H1*). As the majority of the data was collected across the first U.K. national lockdown, it provided a convenient additional test to examine how death thoughts might fluctuate across a lockdown in accordance with COVID-19 case and mortality rates at the time of participation.

To examine this, we utilised open data regarding the number of COVID-19 cases and deaths reported in the U.K. at the time of participation (COVID Index). Our hypothesis was that our COVID Index would positively relate to DTA scores (*H2*). Additionally, we expected that our COVID Index would indirectly predict depression through DTA, for those with low self-esteem (*H3*).

## Method

### Participants

Two-hundred-and-sixty-one participants took part during the first U.K. national lockdown^1^. We excluded 27 participants due to insufficient completed death fragments (<6), and one participant who spent longer than 30 minutes completing the DTA task. The final sample consisted of 233 participants (*M*_age_ = 25.1, SD_age_ = 7.74) with 163 females, 68 males, and two participants identified as non-binary. Ethical approval was obtained from the university.

### Materials and procedure

Participants completed the materials in the following order.^2^

#### Self-esteem

We measured self-esteem with the Rosenberg Self-Esteem scale (Rosenberg, 1965) on a 7-point Likert scale. Higher scores reflect higher self-esteem (α = 0.91).

#### Depression

We measured depression using 7 items from the Depression, Anxiety and Stress Scales – 21 (Lovibond and Lovibond, 1995) on a 4-point Likert scale. Higher scores reflect higher levels of depression (α = 0.89).

#### DTA

We measured DTA using a 33-item word-stem task (Greenberg et al., 1994), 8 of which could be completed in a death-related manner. The word fragments were dead, corpse, skull, mortal, grave, killed, buried, fatal. Higher scores reflect greater DTA (0–8).

#### COVID index

To create our COVID Index, we utilised open data regarding cases and deaths in the United Kingdom on the day of participation (U.K. Health Security Agency, 2021). We logged how many COVID-19 cases and deaths had occurred at the time each participant took part in the study. As case and mortality rates may fluctuate due to changes in reporting methods and other external factors, we opted to take a 3-day rolling average (−1 day to +1 day of participation). We combined case and mortality rates, *r* = 0.95, *p* < 0.001, to create a COVID Index. Higher scores reflect increasing salience (i.e. case and mortality rates) of COVID-19 at the time of participation.

## Results

The means, standard deviations and correlations between the variables are displayed in Table 1. To assess the claim that the extent to which pandemic-induced death thoughts is associated with increased depression when people lack self-esteem (i.e. moderated mediation), we selected Model 14 in PROCESS (Hayes, 2018). This tests for the relationship of an independent variable (COVID Index) on an outcome variable (Depression) through a mediating variable (DTA), with the path between the mediating variable and the outcome variable being moderated by a third variable (Self-Esteem). We standardised the COVID Index, Self-Esteem and DTA scores.

**Table 1.** Means, standard deviations and correlations between the measured variables.

	1.	2.	3.	4.
1. COVID index	–	0.17**	−0.08	0.11^†^
2. DTA		–	−0.28***	0.29***
3. Self-esteem			–	−0.77***
4. Depression				–
Mean	1827.13	3.17	4.60	2.02
SD	774.92	1.51	1.26	.72

*** *p* < 0.001. **p < 0.01. *p < 0.05. ^†^*p* < .10.

In model 1, The COVID Index significantly predicted DTA, β = 0.17, *t* (231) =2.68, *p* = 0.008, 95% CI [0.05, 0.30]. In model 2, There was no significant relationship between the COVID Index, β = 0.03, *t* (228) = 0.99, *p* = 0.323, 95% CI [−.03, 0.09], or DTA, β = 0.04, *t* (228) = 1.32, *p* = 0.189, 95% CI [−.02, 0.10], with depression. Self-esteem was negatively related to depression, β = −0.53 *t* (228) = 16.88, *p* < 0.001, 95% CI [−0.60, −0.47]. The DTA X Self-Esteem interaction was approaching significance, β = −0.06, *t* (228) = 1.86, *p* = 0.065, 95% CI [−0.12, 0.00].

The relationship between DTA and depression was significant at low levels (−1 SD) of Self-Esteem, β = 0.10, *t* (228) = 2.37, *p* = 0.018, 95% CI [0.02, 0.18]. There was no relationship of DTA with depression at high levels (+1 SD) of self-esteem, β = −0.01, *t* (228) = −0.31, *p* = 0.759, 95% CI [–0.11, 0.08].

We examined the indirect effect of the COVID Index on depression through DTA scores at differing levels self-esteem using 5000 bootstrapped samples. The indirect effect of the COVID Index on depression through DTA was significant, Index = −0.01, 95% CI [−0.02, −0.00]. The indirect effect of the COVID Index on depression was significant at low levels of self-esteem, IE = 0.02, 95% CI [0.00, 0.04]. There was no indirect effect of the COVID Index on depression at high levels of self-esteem, IE = −.00, 95% CI [−0.02, 0.01].

## Discussion

Time of participation during a national lockdown was associated DTA. Additionally, these COVID-19-induced death thoughts were associated with increased depression for those with low self-esteem. The present findings carry a number of implications for TMT as well as maintaining sound mental health during a pandemic.

Our study suggests that pandemic-induced death thoughts can undermine sound mental health among those who lack self-esteem. This supports TMT’s view of self-esteem as an essential ingredient to managing death awareness (Juhl, 2019), and provides unique insight into why depression rates have increased. TMT suggests that self-esteem is dependent on living up to culturally valued standards of conduct. Lockdowns have increased financial difficulty, unemployment, and social isolation, which are related to ways in which people may acquire self-esteem. These findings therefore suggest that therapies (Yalom, 2008), and intervention strategies, that appreciate the underlying reasons why people need self-esteem may prove fruitful ways to help individuals maintain sound mental health in a pandemic.

Our findings suggest that existential concerns are heightened in a pandemic, which is a novel finding in the TMT literature. This suggests that behaviours, even those that have no logical connection to mortality, during the pandemic may be driven by existential concerns (Pyszczynski et al., 2021). Additionally, the findings suggest that death thoughts may change temporally across the course of a lockdown, which helps provide a temporal dimension to research considering terror management defences in the context of the COVID-19 pandemic (e.g. Ahmed et al., 2021; Courtney et al., 2021). These findings might help understand why fear drives multiple waves of infection in a pandemic (see Epstein et al., 2008).

More specifically, TMT suggests that when mortality is salient people engage in proximal and distal defences to manage these thoughts (Pyszczynski et al., 1999). Proximal defences can involve reducing one’s perceived susceptibility to health threats, which might increase health-preventative behaviours (e.g. social distancing; Pyszczynski et al., 2021). Proximal defences can also progress to distal defences when they become culturally valued (Courtney et al., 2020). Together proximal and distal defences provide a powerful way to produce adherence to health-preventative behaviours. However, such adherence would wane as the threat of the virus (proximal response), and the emphasis on the cultural value of such behaviours diminishes (distal response) leading to increased transmissibility and a new wave of infection. Future research could examine this idea further by investigating how proximal and distal motivation to engage in health preventative behaviours changes across the course of the pandemic.

The present study is limited because we measured, rather than manipulated, death thoughts, which limits the ability to make strong claims regarding causality. This limitation is inherent to the work conducted because our interest concerned examining how the accessibility of death thoughts can change across the course of a national lockdown. Additionally, measuring death thoughts is recommended when examining constructs that are likely to be resistant to temporary changes such as depression (Cruwys et al., 2015; Fairlamb and Juhl, 2020). Nonetheless, future work could consider manipulating COVID-19 thoughts and measuring DTA and depressive mood, though these would not be able to draw conclusions regarding the temporal effects of death thoughts on depression across the course of a lockdown, which was the focus of this study.

A second limitation concerns the ad-hoc nature to the COVID-19 angle in this work thereby limiting the extent to which we could examine this relationship in more detail. For example, it would have been interesting to know whether self-reported exposure to COVID-19 news, or perceived vulnerability to the virus, might have moderated relationship between the COVID-19 Index and DTA. Death thoughts might not just fluctuate temporally, but this relationship might be dependent on a range of psychological factors.

In sum, the present research highlights the role of death awareness as a neglected factor contributing to rising mental health difficulty worldwide during the pandemic. The findings suggest that the increasing threat of COVID-19 is related to heightened accessibility of death thoughts, which in turn is associated with increased depression for those who lack self-esteem.

## Research Data

sj-docx-1-hpq-10.1177_13591053211067102 – for The relationship between COVID-19-induced death thoughts and depression during a national lockdown Click here for additional data file. sj-docx-1-hpq-10.1177_13591053211067102 for The relationship between COVID-19-induced death thoughts and depression during a national lockdown by Samuel Fairlamb in Journal of Health Psychology This article is distributed under the terms of the
Creative Commons Attribution 4.0 License ( https://creativecommons.org/licenses/by/4.0/)
which permits any use, reproduction and distribution of
the work without further permission provided the original
work is attributed as specified on the SAGE and Open
Access pages ( https://us.sagepub.com/en-us/nam/open-access-at-sage).

sj-sav-2-hpq-10.1177_13591053211067102 – for The relationship between COVID-19-induced death thoughts and depression during a national lockdown Click here for additional data file. sj-sav-2-hpq-10.1177_13591053211067102 for The relationship between COVID-19-induced death thoughts and depression during a national lockdown by Samuel Fairlamb in Journal of Health Psychology This article is distributed under the terms of the
Creative Commons Attribution 4.0 License ( https://creativecommons.org/licenses/by/4.0/)
which permits any use, reproduction and distribution of
the work without further permission provided the original
work is attributed as specified on the SAGE and Open
Access pages ( https://us.sagepub.com/en-us/nam/open-access-at-sage).

sj-sps-3-hpq-10.1177_13591053211067102 – for The relationship between COVID-19-induced death thoughts and depression during a national lockdown Click here for additional data file. sj-sps-3-hpq-10.1177_13591053211067102 for The relationship between COVID-19-induced death thoughts and depression during a national lockdown by Samuel Fairlamb in Journal of Health Psychology This article is distributed under the terms of the
Creative Commons Attribution 4.0 License ( https://creativecommons.org/licenses/by/4.0/)
which permits any use, reproduction and distribution of
the work without further permission provided the original
work is attributed as specified on the SAGE and Open
Access pages ( https://us.sagepub.com/en-us/nam/open-access-at-sage).

sj-spv-4-hpq-10.1177_13591053211067102 – for The relationship between COVID-19-induced death thoughts and depression during a national lockdown Click here for additional data file. sj-spv-4-hpq-10.1177_13591053211067102 for The relationship between COVID-19-induced death thoughts and depression during a national lockdown by Samuel Fairlamb in Journal of Health Psychology This article is distributed under the terms of the
Creative Commons Attribution 4.0 License ( https://creativecommons.org/licenses/by/4.0/)
which permits any use, reproduction and distribution of
the work without further permission provided the original
work is attributed as specified on the SAGE and Open
Access pages ( https://us.sagepub.com/en-us/nam/open-access-at-sage).

## Notes

1. Three hundred thirteen participants in total complete the study, but we limited our analysis to those who took part on, or after Boris Johnson’s announcement to avoid all non-essential travel (16th March) because we wanted to examine how death thoughts may drive depression across the course of a national lockdown. In the interest of transparency, including all participants regardless of time of participation still produces a significant, albeit weaker, relationship between the COVID Index and DTA scores, *r* (283) = 0.13, *p* = 0.033. The indirect effect is not significant, albeit only marginally passes through zero, Index = −0.01, 95% CI [−0.02, 0.00]; IE = 0.01, 95% CI [−0.00, 0.03].

2. The following measures were also included in the survey for exploratory purposes and for brevity do not discuss here: a 3-item stability of self-esteem measure (Altmann and Roth, 2018), a 5-item mania scale (Altman et al., 1997) and the 20-item positive and negative affect schedule (Watson et al., 1988).

## References

[bibr1-13591053211067102] Ahmed R Ahmed A Barkat W (2021) Behavioral limitations of individuals for coping with COVID-19: A terror management perspective. Journal of Human Behavior in the Social Environment 31(1-4): 97–118.

[bibr2-13591053211067102] Altman EG Hedeker D Peterson JL , et al. (1997) The Altman self-rating mania scale. Biological Psychiatry 42(10): 948–955. 9359982 10.1016/S0006-3223(96)00548-3

[bibr3-13591053211067102] Altmann T Roth M (2018) The Self-esteem Stability Scale (SESS) for cross-sectional direct assessment of self-esteem stability. Frontiers in Psychology 9: 91–99. 29487551 10.3389/fpsyg.2018.00091 PMC5816969

[bibr4-13591053211067102] Arndt J Goldenberg JL (2017) Where health and death intersect: Insights from a terror management health model. Current Directions in Psychological Science 26(2): 126–131. 28924332 10.1177/0963721416689563 PMC5600513

[bibr5-13591053211067102] Becker E (1973) The Denial of Death. New York, NY: Free Press.

[bibr6-13591053211067102] Bueno-Notivol J Gracia-García P Olaya B , et al. (2021) Prevalence of depression during the COVID-19 outbreak: A meta-analysis of community-based studies. International Journal of Clinical and Health Psychology 21(1): 100196. 32904715 10.1016/j.ijchp.2020.07.007 PMC7458054

[bibr7-13591053211067102] Burke BL Martens A Faucher EH (2010) Two decades of terror management theory: A meta-analysis of mortality salience research. Personality and Social Psychology Review 14(2): 155–195. 20097885 10.1177/1088868309352321

[bibr8-13591053211067102] Courtney EP Felig RN Goldenberg JL (2021) Together we can slow the spread of COVID-19: The interactive effects of priming collectivism and mortality salience on virus-related health behaviour intentions. British Journal of Social Psychology 61(1): 410–431. 34312892 10.1111/bjso.12487 PMC8420273

[bibr9-13591053211067102] Courtney EP Goldenberg JL Boyd P (2020) The contagion of mortality: A terror management health model for pandemics. British Journal of Social Psychology 59(3): 607–617. 32557684 10.1111/bjso.12392 PMC7323320

[bibr10-13591053211067102] Cruwys T South EI Greenaway KH , et al. (2015) Social identity reduces depression by fostering positive attributions. Social Psychological and Personality Science 6(1): 65–74.

[bibr11-13591053211067102] Das E Bushman BJ Bezemer MD , et al. (2009) How terrorism news reports increase prejudice against outgroups: A terror management account. Journal of Experimental Social Psychology 45(3): 453–459.

[bibr12-13591053211067102] Epstein JM Parker J Cummings D , et al. (2008) Coupled contagion dynamics of fear and disease: Mathematical and computational explorations. PLoS One 3(12): e3955. 10.1371/journal.pone.0003955 PMC2596968 19079607

[bibr13-13591053211067102] Fairlamb S Juhl J (2020) Death thoughts predict increased depression for those with low self-worth. Death Studies. 1–6. DOI: 10.1080/07481187.2020.1793432 32673184

[bibr14-13591053211067102] Gao J Zheng P Jia Y , et al. (2020) Mental health problems and social media exposure during COVID-19 outbreak. PLoS One 15(4): e0231924. 10.1371/journal.pone.0231924 PMC7162477 32298385

[bibr15-13591053211067102] Greenberg J Pyszczynski T Solomon S (1986) The causes and consequences of a need for self-esteem: A terror management theory. In: Baumeister RF (ed.) Public Self and Private Self. New York, NY: Springer, pp.189–212.

[bibr16-13591053211067102] Greenberg J Pyszczynski T Solomon S , et al. (1994) Role of consciousness and accessibility of death-related thoughts in mortality salience effects. Journal of Personality and Social Psychology 67(4): 627–637. 7965609 10.1037//0022-3514.67.4.627

[bibr17-13591053211067102] Hayes AF (2018) Introduction to Mediation, Moderation, and Conditional Process Analysis: A Regression-Based Approach. New York, NY: Guilford Publications.

[bibr18-13591053211067102] Hayes J Schimel J Faucher EH , et al. (2008) Evidence for the DTA hypothesis II: Threatening self-esteem increases death-thought accessibility. Journal of Experimental Social Psychology 44(3): 600–613.

[bibr19-13591053211067102] Hayes J Ward CL McGregor I (2016) Why bother? Death, failure, and fatalistic withdrawal from life. Journal of Personality and Social Psychology 110(1): 96–115. 25915129 10.1037/pspp0000039

[bibr20-13591053211067102] Juhl J (2019) Terror management theory: A theory of psychological well-being. In: Routledge C Vess M (eds) Handbook of Terror Management Theory. Cambridge, MA: Academic Press, pp.303–324.

[bibr21-13591053211067102] Jungmann SM Witthöft M (2020) Health anxiety, cyberchondria, and coping in the current COVID-19 pandemic: Which factors are related to coronavirus anxiety? Journal of Anxiety Disorders 73: 102239. 32502806 10.1016/j.janxdis.2020.102239 PMC7239023

[bibr22-13591053211067102] Lovibond PF Lovibond SH (1995) The structure of negative emotional states: Comparison of the depression anxiety stress scales (DASS) with the Beck depression and anxiety inventories. Behaviour Research and Therapy 33(3): 335–343. 7726811 10.1016/0005-7967(94)00075-u

[bibr23-13591053211067102] Pyszczynski T Greenberg J Solomon S (1999) A dual-process model of defense against conscious and unconscious death-related thoughts: An extension of terror management theory. Psychological Review 106(4): 835–845. 10560330 10.1037/0033-295x.106.4.835

[bibr24-13591053211067102] Pyszczynski T Lockett M Greenberg J , et al. (2021) Terror management theory and the COVID-19 pandemic. Journal of Humanistic Psychology 61(2): 173–189. 10.1177/0022167820959488 PMC7498956 38603072

[bibr25-13591053211067102] Pyszczynski T Solomon S Greenberg J (2015) Thirty years of terror management theory: From genesis to revelation. Advances in Experimental Social Psychology 52: 1–70.

[bibr26-13591053211067102] Rosenberg M (1965) Society and the Adolescent Self-Image. Princeton, NJ: Princeton University Press.

[bibr27-13591053211067102] Routledge C Ostafin B Juhl J , et al. (2010) Adjusting to death: The effects of mortality salience and self-esteem on psychological well-being, growth motivation, and maladaptive behavior. Journal of Personality and Social Psychology 99: 897–916. 21114350 10.1037/a0021431

[bibr28-13591053211067102] Rudenstine S McNeal K Schulder T , et al. (2021) Depression and anxiety during the covid-19 pandemic in an urban, low-income public university sample. Journal of Traumatic Stress 34(1): 12–22. 33045107 10.1002/jts.22600 PMC7675401

[bibr29-13591053211067102] Torales J O’Higgins M Castaldelli-Maia JM , et al. (2020) The outbreak of COVID-19 coronavirus and its impact on global mental health. International Journal of Social Psychiatry 66(4): 317–320. 32233719 10.1177/0020764020915212

[bibr30-13591053211067102] U.K. Health Security Agency (2021) Coronavirus (COVID-19) in the UK. Retrieved May 12, 2021, from: https://coronavirus.data.gov.uk/

[bibr31-13591053211067102] Ustun G (2021) Determining depression and related factors in a society affected by COVID-19 pandemic. International Journal of Social Psychiatry 67(1): 54–63. 32605422 10.1177/0020764020938807 PMC7331110

[bibr32-13591053211067102] Watson D Clark LA Tellegen A (1988) Development and validation of brief measures of positive and negative affect: The PANAS scales. Journal of Personality and Social Psychology 54(6): 1063–1070. 3397865 10.1037//0022-3514.54.6.1063

[bibr33-13591053211067102] Yalom ID (2008) Staring at the Sun: Overcoming the Terror of Death. San Francisco, CA: Jossey-Bass.

