# Supplementary material for: The relationship between COVID-19-induced death thoughts and depression during a national lockdown
Source: J Health Psychol. 2021 Dec 29;27(12):2770–6. doi: 10.1177/13591053211067102 (PMC9537449; doi:10.1177/13591053211067102)
Supplement: sj-docx-1-hpq-10.1177_13591053211067102 – for The relationship between COVID-19-induced death thoughts and depression during a national lockdown [file sj-docx-1-hpq-10.1177_13591053211067102.docx]

The data file includes the filter to recreate the exclusion criteria for the Study. We also present the individual exclusion criteria below:

- DTAfragmentattempts > 5
- Progress > 97
- DTAtasktime < 3600
- Covidindex_3dayav > 325 (i.e., those who took part on the day or after Boris Johnson’s announcement)
